# Associations between dietary inflammatory index and stroke risk: based on NHANES 2005–2018

**DOI:** 10.1038/s41598-024-57267-9

**Published:** 2024-03-20

**Authors:** Ruixian Huang, Fengxia Lai, Le Zhao, Jingjing Zhang, Hao Chen, Shuang Wang, Canjin Chen, Wenhao Wang, Zhenhua Mai, Yuanlin Ding, Danli Kong

**Affiliations:** 1 Department of Epidemiology and Medical Statistics, School of Public Health, Guangdong Medical University, Dongguan, 523808 China; 2 Department of Critical Care Medicine, Affiliated Hospital of Guangdong Medical University, Zhanjiang, 524001 China

**Keywords:** Dietary inflammatory index, Stroke, Insulin resistance, National Health and Nutrition Examination Survey, Mediation analysis, Cardiovascular diseases, Endocrine system and metabolic diseases, Epidemiology, Disease prevention, Nutrition, Public health

## Abstract

The dietary inflammatory index (DII) is a measure of the inflammatory potential of the diet and is closely associated with insulin resistance (IR) and stroke. And IR may play an important role in the development of stroke. Therefore, this study aimed to evaluate the relationship between DII and stroke risk while delving into the potential role of IR in this association. We analyzed data from the National Health and Nutrition Examination Survey (NHANES) from 2005 to 2018, performing weighted univariate analyses, logistic regression, and mediation analyses. At baseline, 3.89% of participants developed stroke, and we observed stroke patients exhibited higher DII scores. After adjusting for covariates, compared to participants in the first quartile of DII scores, those in the third quartile and fourth quartile had increased odds of experiencing a stroke (*OR*: 1.78, 95% *CI*: 1.18–2.68) and (*OR*: 1.70, 95% *CI*: 1.16–2.50), respectively. Moreover, a significant dose–response relationship was observed (*P*-trend < 0.05). However, there was no observed interaction between DII and homeostatic model assessment-IR (HOMA-IR) concerning stroke risk, and HOMA-IR did not mediate the association between DII and stroke. In summary, our study elucidated the significant association between DII and stroke risk, independent of IR. This insight suggests that an anti-inflammatory diet may serve as an effective strategy for stroke prevention.

## Introduction

Stroke is a grave cerebrovascular disorder, ranking as the second leading cause of mortality and the third leading cause of both death and disability globally^1^. With the progressive aging of the population, stroke imposes significant health and economic burdens on individuals and society. Although the precise etiology and mechanisms of stroke remain incompletely elucidated, inflammation unequivocally assumes a central role in its onset and progression^2,3^. Inflammation serves as a natural protective response to bodily injuries or infections; however, sustained, chronic inflammation can lead to endothelial dysfunction, platelet activation, and a prothrombotic state, ultimately heightening the risk of stroke^4^. Additionally, previous studies have indicated that inflammatory mediators such as C-reactive protein (CRP) and interleukin-6 (IL-6) undergo changes during the acute phase of stroke, serving as potential biomarkers for stroke prognosis or imminent complications^5^. In conclusion, inflammation is key in stroke development, holding significant implications for the prevention and management of strokes.

Distinct dietary patterns may result in varying levels of inflammation^6^. Past research has indicated that pro-inflammatory diets can elevate blood concentrations of inflammatory markers such as complement component C3 (C3), CRP, tumor necrosis factor (TNF)-α and IL-6^7^. Therefore, adopting anti-inflammatory diets holds promise as a potential strategy for stroke prevention and intervention. The Dietary Inflammatory Index (DII) is a comprehensive tool used to assess an individual’s dietary inflammation levels based on the impact of various nutrients in the diet on the inflammatory response^8^. While the DII shows promise in dietary studies related to inflammatory diseases, its association with stroke remains poorly understood, and research findings are inconsistent^9,10^. These discrepancies in study outcomes may be attributed to the oversight of underlying factors, such as insulin resistance (IR). IR is a condition characterized by the body's reduced responsiveness to insulin, resulting in ineffective glucose uptake by cells and the development of hyperglycemia^11^. Current research indicates that metabolic changes resulting from inflammation play a crucial role in the development of insulin receptor dysfunction, implying that a more pro-inflammatory diet may elevate the risk of IR^12^. Furthermore, IR is strongly linked not only to diabetes mellitus (DM) and cardiovascular diseases (CVD) but also to the onset and progression of stroke^13,14^. Therefore, IR likely plays a significant role in the interplay between dietary inflammation and stroke.

At present, treatment options for stroke remain very limited^15^. It is crucial to fully understand the risk factors for stroke and take preventive measures to nip it in the bud. There is a complex and subtle relationship between DII, stroke, and IR, but there are no clear research results among these three. Therefore, this study will explore the relationship between DII and stroke in the adult population based on large-scale population data collected in the National Health and Nutrition Examination Survey (NHANES) database, and further investigate the mediating role of IR between the two.

## Results

### Characteristics of participants

The general characteristics of the study population are detailed in Table 1. This study included a total of 13,063 participants with a mean age of 47.41 ± 16.83 years. Among them, 508 individuals were diagnosed with stroke (3.89%). Compared with the non-stroke group, stroke participants exhibited higher proportions of female, non-Hispanic black, education level less than high school, widowed, former drinkers, with underlying conditions (DM, hypertension and hyperlipidemia), and a history of medication use (antidiabetic, antihypertensive, and antihyperlipidemic drugs) (*P* < 0.05). Additionally, the stroke participants had higher levels of age, BMI, FPG, SBP, HOMA-IR and DII, while energy intake, DBP and TC were lower than those in the non-stroke group (*P* < 0.05).

**Table 1:** Characteristics of the study population*.

Variable	Overall (*n* = 13,063)	Non-stroke (*n* = 12,555)	Stroke (*n* = 508)	*χ*^2^/*t*	*P* value
Age	47.41 ± 16.83	46.87 ± 16.64	64.67 ± 13.64	20.79	< 0.001
Gender				4.37	0.039
Female	6508 (0.50)	6248 (0.50)	260 (0.57)		
Male	6555 (0.50)	6307 (0.50)	248 (0.43)		
Race				5.44	0.001
Non-Hispanic Black	2547 (0.11)	2412 (0.10)	135 (0.15)		
Non-Hispanic White	5830 (0.69)	5564 (0.69)	266 (0.70)		
Mexican American	2080 (0.08)	2033 (0.08)	47 (0.04)		
Other Hispanic	1314 (0.05)	1281 (0.05)	33 (0.03)		
Other race	1292 (0.07)	1264 (0.07)	27 (0.07)		
Education level				29.89	< 0.001
Less than high school	3091 (0.15)	2908 (0.15)	183 (0.28)		
Completed high school	3001 (0.23)	2871 (0.23)	130 (0.29)		
More than high school	6971 (0.61)	6776 (0.62)	195 (0.42)		
Married				30.71	< 0.001
Never married	2289 (0.18)	2251 (0.18)	38 (0.05)		
Partner or married	7960 (0.64)	7673 (0.64)	287 (0.64)		
Separated or divorced	1836 (0.12)	1739 (0.12)	97 (0.16)		
Widowed	978 (0.05)	892 (0.05)	86 (0.15)		
Drinking status				52.52	< 0.001
Never	1757 (0.11)	1675 (0.10)	82 (0.15)		
Former drinker	2152 (0.13)	1978 (0.13)	174 (0.32)		
Current drinker	9154 (0.76)	8902 (0.77)	252 (0.53)		
BMI	29.01 ± 6.89	28.96 ± 6.87	30.41 ± 7.46	2.69	0.008
Energy intake	2094.46 ± 774.877	2103.01 ± 774.44	1819.33 ± 738.09	− 5.94	< 0.001
FPG	5.89 ± 1.64	5.87 ± 1.61	6.57 ± 2.27	4.85	< 0.001
Insulin	12.58 ± 14.74	12.53 ± 14.70	14.15 ± 15.89	1.96	0.052
SBP	121.53 ± 16.85	121.26 ± 16.63	130.17 ± 20.89	7.55	< 0.001
DBP	69.68 ± 12.05	69.76 ± 11.93	66.87 ± 15.01	− 3.29	0.001
TC	4.99 ± 1.07	4.99 ± 1.06	4.81 ± 1.19	− 2.60	0.011
HDL-C	1.40 ± 0.42	1.4 ± 0.42	1.38 ± 0.45	− 0.72	0.474
HOMA-IR	3.54 ± 5.79	3.51 ± 5.76	4.56 ± 6.74	2.56	0.012
DII	1.43 ± 1.86	1.41 ± 1.86	2.01 ± 1.68	7.14	< 0.001
Quartiles of HOMA-IR				7.37	< 0.001
Q1	3267 (0.28)	3165 (0.28)	102 (0.25)		
Q2	3265 (0.26)	3156 (0.26)	109 (0.20)		
Q3	3265 (0.24)	3138 (0.24)	127 (0.23)		
Q4	3266 (0.22)	3096 (0.22)	170 (0.32)		
Quartiles of DII				8.96	< 0.001
Q1	3266 (0.27)	3185 (0.27)	81 (0.17)		
Q2	3266 (0.26)	3147 (0.26)	119 (0.23)		
Q3	3265 (0.24)	3126 (0.24)	139 (0.29)		
Q4	3266 (0.23)	3097 (0.23)	169 (0.31)		
DM				99.88	< 0.001
No	10,319 (0.85)	10,032 (0.85)	287 (0.63)		
Yes	2744 (0.16)	2523 (0.15)	221 (0.37)		
Hypertension				219.98	< 0.001
No	7492 (0.62)	7394 (0.64)	98 (0.23)		
Yes	5571 (0.38)	5161 (0.36)	410 (0.77)		
Hyperlipidemia				61.31	< 0.001
No	3590 (0.29)	3532 (0.30)	58 (0.10)		
Yes	9473 (0.71)	9023 (0.70)	450 (0.90)		
Anti-diabetic drugs				100.05	< 0.001
No	11,525 (0.91)	11,160 (0.92)	365 (0.76)		
Yes	1538 (0.09)	1395 (0.08)	143 (0.24)		
Anti-hypertension drugs				331.02	< 0.001
No	8820 (0.72)	8695 (0.73)	125 (0.28)		
Yes	4243 (0.28)	3860 (0.27)	383 (0.72)		
Anti-hyperlipidemic drugs				309.03	< 0.001
No	10,328 (0.81)	10,098 (0.82)	230 (0.43)		
Yes	2735 (0.19)	2457 (0.18)	278 (0.57)		

*Percentage and mean ± standard deviation were weighted. *t*-test was used for continuous variable and *χ*^2^ test was used for categorical variables.

### Characteristics of the participants according to the quartiles of DII

The characteristics of participants by DII quartile are presented in Table 2. With increasing DII scores, participants tend to exhibit higher levels of BMI, insulin, SBP, TC, and HOMA-IR, while displaying lower levels of energy intake, DBP, and HDL-C (*P* < 0.05). Furthermore, we observed that, in comparison to the first quartile of DII, participants in the second through fourth quartiles of DII showed a higher prevalence of female, non-Hispanic blacks, stroke, with underlying conditions (DM, hypertension and hyperlipidemia), and a history of antihypertensive drugs use. Conversely, the proportion of participants with an education level of more than high school, partner or married, and current drinkers was lower (*P* < 0.05). The characteristics of participants according to the DII tertiles were similar to those of DII quartiles, except HDL-C, HOMA-IR and antidiabetic drugs. Based on the DII tertiles, when compared to the first tertiles, individuals in the second tertiles and third tertiles had higher a history of antidiabetic drugs use, but the clinical correlation between DII tertiles and HDL-C and HOMA-IR has not been definitively established (*P* > 0.05) (Supplementary Table S1).

**Table 2:** Characteristics of the participants according to the quartiles of DII*.

Variable	Quartiles of DII				*χ*^2^/*t*	*P* value
	Q1	Q2	Q3	Q4		
Age	48.05 ± 15.97	47.38 ± 16.67	47.00 ± 17.05	47.11 ± 17.75	− 1.73	0.086
Gender					109.45	< 0.001
Female	1245 (0.39)	1477 (0.46)	1747 (0.55)	2039 (0.64)		
Male	2021 (0.611)	1789 (0.54)	1518 (0.45)	1227 (0.36)		
Race					8.75	< 0.001
Non-Hispanic Black	474 (0.07)	569 (0.09)	716 (0.12)	787 (0.14)		
Non-Hispanic White	1532 (0.72)	1470 (0.69)	1389 (0.67)	1439 (0.67)		
Mexican American	566 (0.09)	540 (0.08)	524 (0.08)	450 (0.07)		
Other Hispanic	295 (0.05)	337 (0.06)	343 (0.05)	339 (0.06)		
Other race	398 (0.07)	350 (0.07)	293 (0.07)	251 (0.06)		
Education level					31.69	< 0.001
Less than high school	592 (0.11)	720 (0.14)	827 (0.17)	952 (0.20)		
Completed high school	618 (0.18)	705 (0.21)	780 (0.25)	898 (0.30)		
More than high school	2056 (0.71)	1841 (0.65)	1658 (0.58)	1416 (0.50)		
Married					8.85	< 0.001
Never married	540 (0.17)	521 (0.17)	611 (0.19)	617 (0.19)		
Partner or married	2163 (0.70)	2075 (0.67)	1920 (0.62)	1802 (0.59)		
Separated or divorced	380 (0.10)	451 (0.12)	473 (0.13)	532 (0.15)		
Widowed	183 (0.04)	219 (0.05)	261 (0.06)	315 (0.07)		
Drinking status					18.45	< 0.001
Never	350 (0.08)	403 (0.10)	451 (0.11)	553 (0.14)		
Former drinker	4399 (0.11)	487 (0.12)	556 (0.14)	670 (0.17)		
Current drinker	2477 (0.81)	2376 (0.78)	2258 (0.75)	2043 (0.69)		
BMI	28.30 ± 6.55	28.85 ± 6.64	29.33 ± 7.22	29.68 ± 7.12	5.60	< 0.001
Energy intake	2554.69 ± 799.13	2203.72 ± 689.36	1944.75 ± 639.05	1585.33 ± 592.37	− 26.78	< 0.001
FPG	5.83 ± 1.54	5.93 ± 1.73	5.88 ± 1.63	5.91 ± 1.65	1.66	0.100
Insulin	11.43 ± 10.61	13.05 ± 19.85	13.01 ± 13.67	12.94 ± 13.17	4.19	< 0.001
SBP	120.70 ± 16.12	121.42 ± 16.29	122.11 ± 16.74	122.02 ± 18.31	2.65	0.009
DBP	70.35 ± 11.37	70.12 ± 11.79	69.66 ± 12.377	68.40 ± 12.65	− 3.05	0.003
TC	4.93 ± 1.03	5.01 ± 1.04	5.04 ± 1.09	4.97 ± 1.11	2.56	0.012
HDL-C	1.42 ± 0.42	1.41 ± 0.44	1.41 ± 0.42	1.38 ± 0.41	− 2.26	0.026
HOMA-IR	3.17 ± 4.14	3.78 ± 7.91	3.62 ± 4.63	3.65 ± 5.75	3.17	0.002
Quartiles of HOMA-IR					4.38	< 0.001
Q1	932 (0.32)	828 (0.28)	766 (0.27)	741 (0.24)		
Q2	836 (0.26)	831 (0.26)	801 (0.24)	797 (0.26)		
Q3	791 (0.22)	793 (0.24)	850 (0.26)	831 (0.25)		
Q4	707 (0.19)	814(0.22)	848 (0.23)	897 (0.25)		
Stroke					8.96	< 0.001
No	3185 (0.98)	3147 (0.97)	3126 (0.96)	3097 (0.96)		
Yes	81 (0.02)	119 (0.03)	139 (0.04)	169 (0.04)		
DM					7.32	< 0.001
No	2685 (0.87)	2596 (0.85)	2524 (0.83)	2514 (0.82)		
Yes	581 (0.13)	670 (0.15)	741 (0.17)	752 (0.18)		
Hypertension					3.34	0.023
No	1948 (0.64)	1915 (0.63)	1882 (0.63)	1747 (0.59)		
Yes	1318 (0.36)	1351 (0.37)	1383 (0.37)	1519 (0.41)		
Hyperlipidemia					4.74	0.003
No	1014 (0.32)	882 (0.28)	879 (0.28)	815 (0.27)		
Yes	2252 (0.68)	2384 (0.72)	2386 (0.72)	2451 (0.73)		
Antidiabetic drugs					1.94	0.127
No	2939 (0.92)	2883 (0.91)	2854 (0.91)	2849 (0.90)		
Yes	327 (0.08)	383 (0.09)	411 (0.09)	417 (0.10)		
Antihypertensive drugs					3.17	0.028
No	2291 (0.74)	2209 (0.72)	2195 (0.73)	1125 (0.70)		
Yes	975 (0.26)	1057 (0.28)	1070 (0.27)	1141 (0.30)		
Antihyperlipidemic drugs					0.89	0.440
No	2580 (0.80)	2599 (0.82)	2590 (0.81)	2559 (0.81)		
Yes	686 (0.20)	667 (0.18)	675 (0.19)	707 (0.19)		

*Percentage and mean ± standard deviation were weighted. The linear regression was used for continuous variable and *χ*^2^ test was used for categorical variables.

### Association between DII and stroke

**Figure 1:**
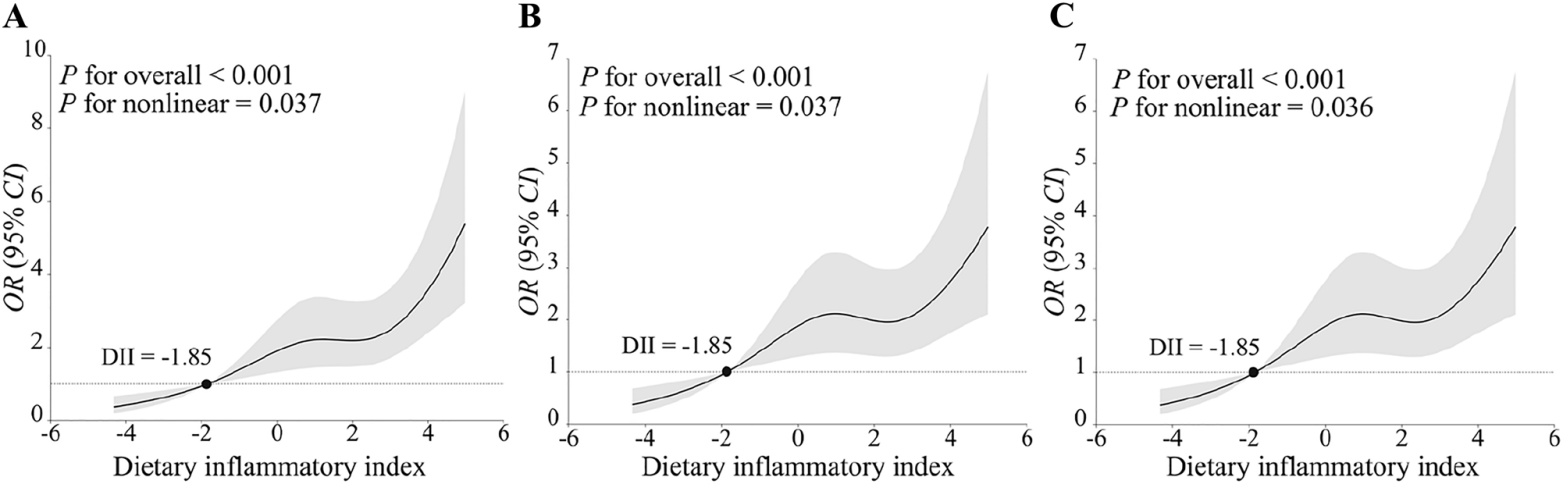
The restricted cubic spline curve was used to model the relationship between DII and the risk of stroke among all participants (**A**–**C**). (**A**) did not adjust any covariates; (**B**) adjusted for age, gender, race, education, marital status, BMI, energy intake, drinking status, FPG, SBP, DBP, TC, HDL-C, DM, hypertension, hyperlipidemia, antidiabetic drugs, antihypertensive drugs, and antihyperlipidemic drugs; (**C**) further adjusted for HOMA-IR.

As shown in Table 3, the DII demonstrated a positive correlation with the risk of stroke in the second quartile [*OR*: 1.45 (95% *CI*: 1.02–2.08)] to the fourth quartile [*OR*: 2.20 (95% *CI*: 1.63–2.95)] compared with the first quartile in Model 1. This positive correlation persisted in the third quartile [*OR*: 1.78 (95% *CI*: 1.18–2.68) and the fourth quartile [*OR*: 1.70 (95% *CI*: 1.16–2.50)] compared with the first quartile in Model 2 and Model 3. Importantly, a significant dose–response relationship was evident in all three models (*P* < 0.05). An analysis with DII to increase 1-SD yielded similar results in Model 1 [*OR*: 1.42 (95% *CI*: 1.27–1.58)], Model 2 and Model 3 [*OR*: 1.30 (95% *CI*: 1.13–1.51)]. The spline variable confirmed that DII in all three models were significant non-linearly associated with the risk of stroke (*P*-nonlinear < 0.05), and the graph of the relationship revealed that an increase in DII was consistently associated with an increased risk of stroke (shown in Fig. 1). Similarly, the association between DII tertiles and stroke displayed the same characteristics. (Supplementary Table S2).

**Table 3:** Risk of stroke according to quartiles of DII.

	Model 1^a^		Model 2^b^		Model 3^c^	
	*OR* (95% *CI*)	*P* value	*OR* (95% *CI*)	*P* value	*OR* (95% *CI*)	*P* value
Quartiles of DII						
Q1	Ref. (1.00)		Ref. (1.00)		Ref. (1.00)	
Q2	1.45 (1.02–2.08)	0.040	1.40 (0.95–2.06)	0.083	1.41 (0.95–2.08)	0.089
Q3	1.96 (1.41–2.73)	< 0.001	1.78 (1.18–2.67)	0.006	1.78 (1.18–2.68)	0.006
Q4	2.20 (1.63–2.95)	< 0.001	1.70 (1.16–2.49)	0.007	1.70 (1.16–2.50)	0.007
*P*-trend	< 0.001		0.006		0.006	
For 1-SD increase	1.42 (1.27–1.58)	< 0.001	1.30 (1.13–1.51)	< 0.001	1.30 (1.13–1.51)	< 0.001

^a^Model 1: Did not adjust any covariates;

^b^Model 2: Adjusted for age, gender, race, education, marital status, BMI, energy intake, drinking status, FPG, SBP, DBP, TC, HDL-C, DM, hypertension, hyperlipidemia, antidiabetic drugs, antihypertensive drugs, and antihyperlipidemic drugs;

^c^Model 3: Further adjusted for HOMA-IR.

### DII and stroke risk stratified by HOMA-IR category

Table 4 reveals that when stratified by HOMA-IR category, the risk of DII and stroke was primarily notable in the fourth quartile of DII except for individuals with 1.50 ≤ HOMA-IR < 2.51 in Model 1 [the *OR*s (95% *CI*s) were 2.89 (1.45–5.75), 2.02 (1.11–3.67), 2.22 (1.22–4.05)]. And in Model 2, the risk of DII and stroke was primarily notable in the fourth quartile of DII for individuals with 0.03 ≤ HOMA-IR < 1.50 [the *OR* (95% *CI*) was 2.87 (1.04–7.89)]. Additionally, there was no interaction observed between DII and HOMA-IR on stroke risk (*P-*interaction > 0.05). The risk of DII tertiles and stroke displayed similar characteristics excluding the association between them in Model 2 (Supplementary Table S3).

**Table 4:** Risk of stroke by quartile of dietary inflammatory index stratified according to HOMA-IR.

Variable	Participants/event	Model 1^a^	Model 2^b^
		OR (95% CI)	OR (95% CI)
Quartiles of DII			
0.03 ≤ HOMA-IR < 1.50			
Q1	932/20	Ref. (1.00)	Ref. (1.00)
Q2	828/22	2.00 (0.92–4.35)	2.19 (0.89–5.40)
Q3	766/23	2.50 (1.04–6.01)	2.37 (0.80–7.01)
Q4	741/37	2.89 (1.45–5.75)	2.87 (1.04–7.89)
*P*-trend		0.001	0.045
1.50 ≤ HOMA-IR < 2.51			
Q1	836/18	Ref. (1.00)	Ref. (1.00)
Q2	831/28	0.88 (0.41–1.89)	0.93 (0.39–2.20)
Q3	801/26	1.65 (0.77–3.58)	1.74 (0.72–4.18)
Q4	797/37	1.47 (0.71–3.03)	1.24 (0.50–3.07)
*P*-trend		0.123	0.397
2.51 ≤ HOMA-IR < 4.36			
Q1	791/21	Ref. (1.00)	Ref. (1.00)
Q2	793/28	1.49 (0.69–3.21)	1.45 (0.66–3.16)
Q3	850/41	2.05 (1.07–3.91)	1.71 (0.85–3.44)
Q4	831/37	2.02 (1.11–3.67)	1.55 (0.74–3.26)
*P*-trend		0.007	0.213
HOMA-IR ≥ 4.36			
Q1	707/22	Ref. (1.00)	Ref. (1.00)
Q2	814/41	1.43 (0.70–2.95)	1.33 (0.63–2.79)
Q3	848/49	1.64 (0.81–3.29)	1.58 (0.79–3.19)
Q4	897/58	2.22 (1.22–4.05)	1.83 (0.99–3.37)
*P*-trend		0.005	0.046
*P*_*interaction*_		0.872	0.571

^a^Model 1: Did not adjust any covariates;

^b^Model 2: Adjusted for age, gender, race, education, marital status, BMI, energy intake, drinking status, FPG, SBP, DBP, TC, HDL-C, DM, hypertension, hyperlipidemia, antidiabetic drugs, antihypertensive drugs, and antihyperlipidemic drugs.

### Mediating role of HOMA-IR

**Figure 2:**
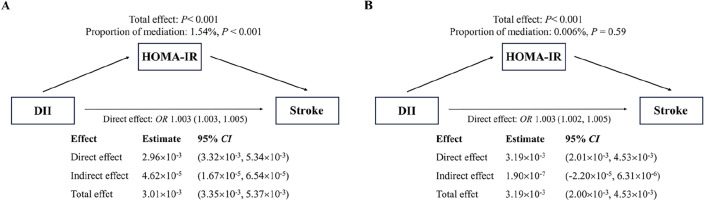
Mediating effect of HOMA-IR between DII (**A**,**B**) and stroke. The 95% *CI* of these estimates was computed using the bootstrap method (1000 samples). (**A**) did not adjust any covariates; (**B**) adjusted for age, gender, race, education, marital status, BMI, energy intake, drinking status, FPG, SBP, DBP, TC, HDL-C, DM, hypertension, hyperlipidemia, antidiabetic drugs, antihypertensive drugs, and antihyperlipidemic drugs.

The result of the mediation analysis is shown in Fig. 2. Increased DII was associated with an increased risk of stroke, and the effect (1.54%) can be explained by a significant indirect effect of HOMA-IR (*OR*: 4.62 × 10^−5^, 95% *CI*: 1.67 × 10^−5^ to 6.54 × 10^−5^) (Fig. 2A). After adjusting for covariates, the indirect effect was not statistically significant (*OR*: 1.90 × 10^−7^, 95% *CI*: − 2.20 × 10^−5^ to 6.31 × 10^−6^) (Fig. 2B).

## Discussion

In this cross-sectional observational study, a total of 13,063 participants were recruited to assess the correlation between DII and IR with the risk of stroke. Our results indicate that DII is an independent risk factor for stroke in the adult population of the U.S. The baseline inflammatory characteristics of diet show a positive non-linear association with the risk of stroke, and this association remains significant even after adjusting for known or potential confounding factors. Furthermore, we observed that stroke patients often have higher HOMA-IR levels, which gradually increase with higher DII scores. In Model 1 (Fig. 2A), which did not adjust for relevant covariates, HOMA-IR played a weaker mediating role in the association between DII and stroke. However, when considering these covariates (Fig. 2B), HOMA-IR no longer mediated the relationship between DII and stroke. Finally, there is insufficient evidence to support a substantial interaction between DII and HOMA-IR on the risk of stroke.

Prior studies have primarily centered on exploring the link between DII and CVD. In the Ravansar Non-Communicable Disease (RaNCD) cohort study^16^, where 9% of participants had prior CVD records, the investigation set out to scrutinize the connection between DII and CVD risk. The findings unveiled that higher adherence to a pro-inflammatory diet correlated with an elevated risk of CVD (*OR*: 1.4, 95% *CI*: 1.1–1.8). In a large prospective cohort study, the Supplémentation en VItamines et Minéraux AntioXydants (SU.VI.MAX)^17^, the investigation focused on the correlation between DII and the occurrence of overall CVD and its subtypes, including myocardial infarction, stroke, sudden death, angina pectoris and revascularization interventions. The results showed a noteworthy connection between a pro-inflammatory dietary pattern and an elevated risk of myocardial infarction, while such an association was not observed for the other subtypes of CVD. Furthermore, the study by Garcia-Arellano^18^ and Ramallal^19^ et al. also demonstrated that the quartiles of DII were positively associated with the overall risk of CVD and exhibited a linear dose–response trend.

In this study, we specifically focused on the subtype of CVD, which is stroke. Our findings revealed that the DII scores were higher in the stroke group as opposed to the non-stroke group (Table 1). Participants with higher DII scores (reflecting a pro-inflammatory diet) had a greater risk of stroke in comparison to those with lower DII scores (reflecting an anti-inflammatory diet) (Table 3). This observation corresponds with previous research on the relationship between DII and stroke^10,20,21^. Shi^10^ and Ganbaatar^20^ study suggest that individuals who have had a stroke tend to follow diets with higher inflammatory potential, and those with elevated DII scores face a higher risk of stroke. Comparing the fourth quartile with the first quartile, the risk of stroke was reported as (*HR*: 1.34, 95% *CI*: 1.03–1.75) and (*OR*: 1.21, 95% *CI*: 1.06–1.38). However, their study exclusively considered a single division method for DII, which might not adequately account for the potential for false positive or false negative results due to interval interference. Similarly, in a case–control study, it was observed that adopting a healthy dietary pattern played a significant role in reducing the risk of stroke when compared to the control group^21^. But their study had a relatively small sample size, highlighting the need for improving sample representativeness. In contrast, our study not only utilized data from a large and diverse sample of individuals but also conducted a thorough analysis utilizing two division methods (quartiles and tertiles), significantly enhancing the representativeness of the sample and the reliability of the data results.

However, it is also inconsistent with the findings of some studies. The SU.VI.MAX cohort^17^ and a prospective cohort study of Australian women^22^ both reported a lack of statistical association between DII scores and stroke. We posit several potential reasons for these findings. Firstly, the prospective cohort study may have had a limited number of participants in the stroke group, leading to reduced sample robustness. Secondly, these studies encompassed diverse groups with variations in racial backgrounds, dietary practices, and cultural norms. Our investigation was centered on the American population, while the two prospective cohorts predominantly examined French and Australian populations. Last but not the least, variations were evident in both the age range and gender composition of participants across the different studies. Our study exclusively involved American adults aged 20 years and older, whereas the SU.VI.MAX cohort enrolled individuals aged 35 to 60, and the Australian cohort concentrated on females aged 50 to 55.

In our study, stroke patients had higher HOMA-IR levels (Table 1). Compared to the first quartile, the second through fourth quartiles also showed elevated HOMA-IR levels (Table 2). Bienek et al. made similar observations, noting increased insulin concentrations and HOMA-IR levels in stroke patients^23^. Many studies have confirmed the relationship between stroke and IR. According to Rundek et al., IR serves as a marker for an increased risk of stroke in non-diabetic patients^24^. Research from a Japanese community-based cohort study also identified elevated HOMA-IR levels as a significant risk factor for stroke, even after adjusting for covariates (*HR*: 1.47, 95% *CI*: 1.14–1.88)^25^. The relationship between DII and IR is a subject of ongoing debate among scholars. While some studies, like that of Moslehi et al., found no significant association between DII and insulin resistance in Iranian adults^26^. others, such as Shu et al., strongly argue for a close relationship between DII and IR, showing that higher DII scores are associated with larger HOMA-IR values^12^. Furthermore, a meta-analysis also supports the strong association between IR and DII^27^. Our study partly validates this association, but a limitation arises when categorizing DII into tertiles. While HOMA-IR levels increase with higher DII scores, the clinical significance remains unclear (Supplementary Table S1). This could be related to data distribution and variability, as quartiles for DII may better capture the association with IR. It's also possible that a threshold effect exists, with a significant increase in IR observed only when DII values exceed a certain threshold. Current research suggests that inflammation plays a pivotal role in causing pancreatic dysfunction and elevating circulating fatty acid levels, contributing to the occurrence and development of cardiovascular diseases^28,29^. Nonetheless, our study has uncovered that the link between DII and stroke may operate independently of IR. The HOMA-IR scores (ranging from 0.028 to 269.41) were analyzed in quartiles (Supplementary Table S4). In comparison to the threshold-based method (HOMA-IR = 2.7)^30^, the quartile approach provides a more comprehensive exploration of insulin resistance at different levels, capturing potential diversity and variability beyond specific threshold values. It is noteworthy that even after adjusting for HOMA-IR, a significant association between DII and stroke persists (Table 3). Moreover, within our stratified model, an increased stroke risk was evident exclusively within the Q3, Q4, T2, or T3 DII ranges for particular HOMA-IR intervals, with no observable interaction between DII and HOMA-IR. This implies that HOMA-IR and DII may independently impact the occurrence of stroke. In the mediation analysis, HOMA-IR exhibited weak mediation in the unadjusted model (Fig. 2A) and became insignificant after multivariate adjustment (Fig. 2B). This implies that other factors may influence the relationship between DII and stroke regarding the effect of HOMA-IR. Subsequent research could consider potential mediators among the adjusted covariates to further investigate their role in the connection between DII and stroke.

This study exhibits multiple strengths. Firstly, we leveraged extensive, nationally representative sample data and meticulously accounted for potential confounding factors, rendering the results both robust and credible. Secondly, a diverse range of analytical methods were employed to investigate the correlation between DII and stroke, as well as the influence of IR on it. Our study elucidated the independent association of DII with the risk of stroke, and it demonstrated that IR and DII did not exhibit synergistic effects on the risk of the disease, a finding that holds significant public health implications.

Nonetheless, it’s important to acknowledge certain limitations in our interpretation of the results. Firstly, our DII score estimation was based on just 27 dietary parameters. It’s worth noting that no previous study has employed all 45 dietary parameters for calculating DII scores concerning stroke risk or even CVD risk. In previous studies, most studies used only 20–36 dietary parameters to calculate DII scores^17,20,31–33^. Moreover, one study has reported that a reduction in the number of dietary parameters available did not significantly impact the predictive capability of DII scores^34^. Additionally, information about stroke events was obtained through individual interviews, potentially introducing data collection bias due to the presence of severe stroke patients who were not equipped to respond to interviews. Furthermore, NHANES is a cross-sectional study, making it unsuitable for determining causality. To add further, dietary intake was estimated based on a 24-h recall, introducing potential recall bias that may not accurately reflect individuals' daily dietary habits. However, some studies suggest that the 24-h recall method may be sufficient to assess an individual's daily dietary intake^35^. Despite these limitations, our study contributes valuable insights into the association between DII, IR, and stroke. Further prospective studies are necessary to establish precise associations between variables and the occurrence of stroke.

In summary, our study indicates that as the DII increases, it is associated with elevated levels of indices related to IR. Furthermore, DII is independently linked to an increased risk of stroke in American adults. We found no sufficient evidence to support a multiplicative effect of DII and HOMA-IR on disease risk, nor did we find evidence that HOMA-IR acts as a mediator in this relationship.

## Methods

### Participants and study design

**Figure 3:**
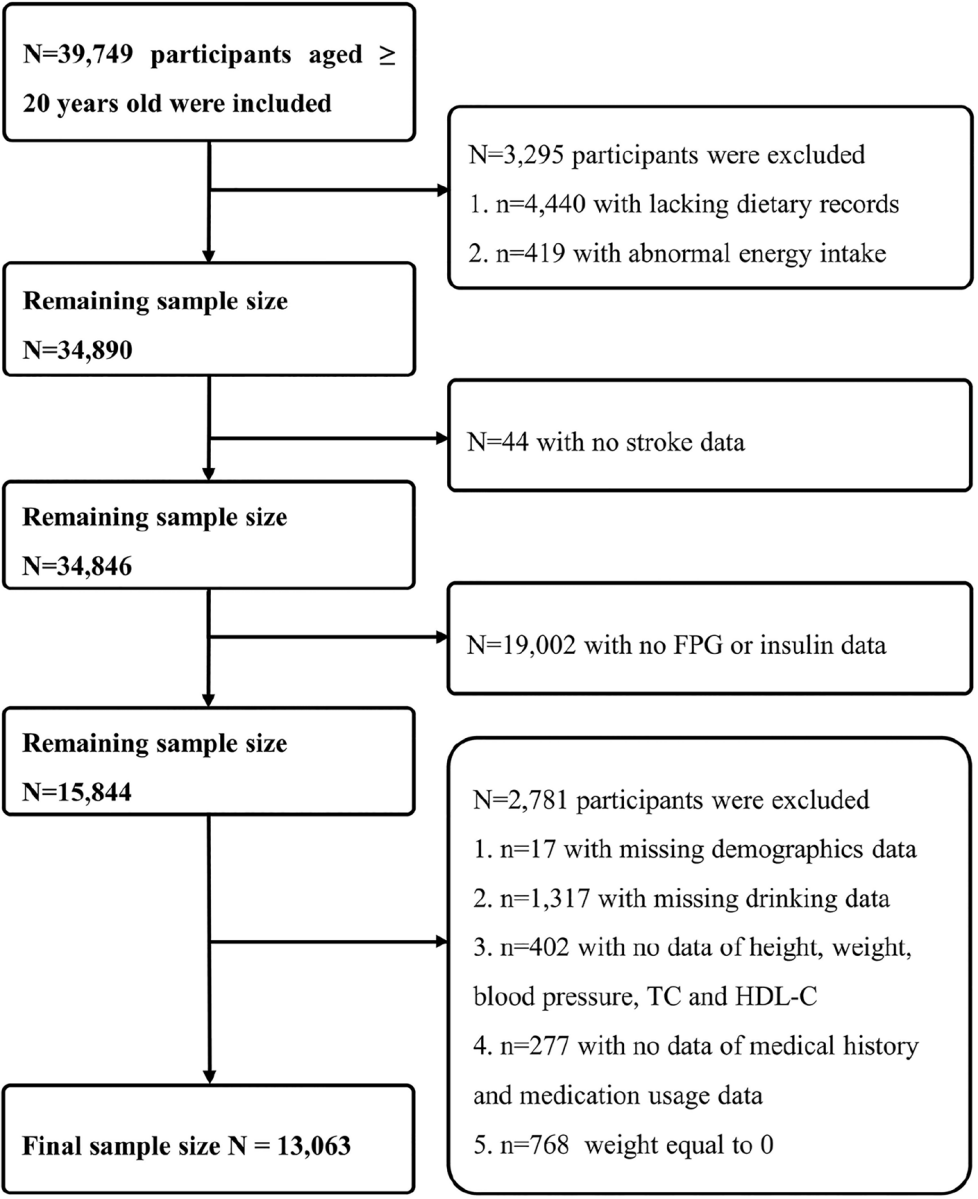
Flowchart of the participants selection from NHANES 2005–2018.

NHANES is a cross-sectional survey in the United States that integrates interviews and physical examinations. Its primary objective is to gather data regarding the health and nutritional status of both adults and children. NHANES uses a complex, stratified, multistage probabilistic study design to recruit a representative sample of the U.S. population annually and obtain informed consent from all participants. We employed NHANES data encompassing seven survey cycles spanning from 2005 to 2018. Initially, there were a total of 70,190 participants in our study. However, we restricted our analysis to 39,749 subjects aged 20 years and older. We excluded individuals based on the following criteria: (i) lacking dietary records or abnormal energy intake (daily energy intake ≤ 500 kcal or ≥ 5000 kcal) (n = 3295); (ii) without stroke data (n = 44); (iii) without fasting plasma glucose (FPG) or insulin value (n = 19,002); (iv) without demographics [gender, education level, race and marital status] or drinking data or physical examination data [height weight and blood pressure] or laboratory tests data [total cholesterol (TC) and high density lipoprotein cholesterol (HDL-C)] or medical history data [DM, hypertension, hyperlipidemia, as well as the use of antidiabetic, antihypertensive, and antihyperlipidemic drugs] or weight equal to 0 (n = 2781). Finally, we arrived at a final dataset comprising 13,063 participants for our comprehensive statistical analysis (Fig. 3).

### Dietary inflammatory index

DII is a comprehensive tool primarily designed to assess an individual's overall dietary inflammatory potential. It utilizes data derived from single 24-h dietary recalls within the NHANES dataset, which includes 27 dietary ingredients and nutrient parameters used for calculating the DII score^10,31^. These components comprise carbohydrates, protein, cholesterol, various types of fats (saturated, monounsaturated, polyunsaturated), omega-3 and omega-6 polyunsaturated fatty acids, vitamins (A, B1, B2, B6, B12, C, D, E), niacin, folic acid, beta carotene, minerals (iron, magnesium, zinc, selenium), alcohol, fiber, and caffeine. The DII calculation involves several key steps^31^. Initially, the intake of each dietary ingredient or nutrient is standardized using Z-scores. This process entails subtracting the individual’s estimated intake from the standard average and then dividing it by the world standard deviation, resulting in a distribution centered at 0 and bounded between − 1 and + 1. Subsequently, the Z-scores are multiplied by each dietary component’s respective inflammatory effect score. The summation of these calculations yields the individual’s DII score, which can range from negative values (indicating anti-inflammatory properties) to positive values (indicating pro-inflammatory properties).

### Stroke

We defined stroke based on self-reported interview data obtained from the Medical Condition Questionnaire. The participants were asked the following questions: “Has a doctor or other health professional ever told you that you have had a stroke?” If they answered in the affirmative, they were categorized into the stroke group. Conversely, participants who denied having been informed of a stroke were included in the non-stroke group^10^.

### Homeostatic model assessment of insulin resistance

The homeostatic model assessment of insulin resistance (HOMA-IR) was employed to evaluate IR levels in individuals. HOMA-IR is calculated as the product of fasting insulin (μU/mL) and fasting blood glucose (mmol/L), divided by 22.5^30^. A HOMA-IR value of 1 is considered normal, with higher values indicating a greater degree of IR in individuals. IR was defined as a HOMA-IR value exceeding 2.7^30^.

### Study covariates

Drawing from previous research, multivariable models have taken into account potential confounding variables associated with the relationship between DII and stroke^10,12,24,36,37^. These variables encompass the following factors: age, gender (female or male), race (non- Hispanic black, non-Hispanic white, Mexican American, other Hispanic, other race), education level (less than high school, completed high school and more than high school), marital status (never married, partner or married, separated and divorced, widowed), body mass index (BMI), energy intake, drinking status (never, former drinker, current drinker), FPG, systolic blood pressure (SBP), diastolic blood pressure (DBP), TC, HDL-C, DM, hypertension, hyperlipidemia, and the use of antidiabetic, antihypertensive, and antihyperlipidemic drugs.

### Statistical analysis

We weighted the NHANES data for analysis. Baseline population characteristics were reported as mean ± standard deviation for continuous variables and as numerical values (percentage) for categorical variables. Weighted Student’s *t*-test or weighted linear regression was employed to analyze differences among continuous variables. The weighted chi-squared test was used to assess disparities in categorical variables. Then, to estimate the association between stroke and DII quartiles, logistic regression was employed in three different models. We calculated the odds ratio (*OR*) and 95% confidence interval (*CI*) for each model, reporting the risk of stroke occurrence for each 1-SD (z-score) increase in DII. Three models were proposed: Model 1 without any adjustment for confounding factors; Model 2 adjusted for age, gender, race, education, marital status, BMI, energy intake, drinking status, FPG, SBP, DBP, TC, HDL-C, DM, hypertension, hyperlipidemia, and the use of antidiabetic, antihypertensive, and antihyperlipidemic drugs; Model 3 further adjusted for HOMA-IR. Furthermore, in order to evaluate whether there is a dose–response relationship between DII and stroke among the three models, we placed three nodes at the 5th, 35th, 65th and 95th percentiles of the DII distribution to construct restricted cubic spline models.

We conducted a stratified analysis in Models 1 and 2 by dividing HOMA-IR into quartiles to evaluate its potential moderating impact on IR. We also assessed the impact of the interaction between DII and HOMA-IR on stroke by introducing a multiplicative term between the two variables within a logistic model. Afterward, we employed mediation models using both linear and logistic regression to assess the direct impact of DII on stroke risk and the indirect influence mediated by HOMA-IR. Three effect estimates were obtained through the bootstrap method: the total effect, representing the overall effect of DII on stroke; the direct effect, indicating the effect of DII on stroke after controlling for the mediator variable HOMA-IR; and the indirect effect, indicating the effect of DII on stroke mediated by HOMA-IR. In addition, we conducted sensitivity analyses by reclassifying DII into tertiles to mitigate the impact of different DII categorizations on the results. All statistical analyses were conducted with R version 4.2.3 (https://www.r-project.org/, The R Foundation), and the “survey” packages was used. A significance level of *P*-values < 0.05 (two-tailed) was considered statistically significant.

### Ethics statement

The NHANES protocols were approved by the ethics review board and included the written informed consent of all participants, following the principles of the Declaration of Helsinki.

### Supplementary Information

Supplementary Tables.

## Data Availability

The data supporting the findings of this study can be obtained from NHANES (https://www.cdc.gov/nchs/nhanes/index.htm).

## Abbreviations

BMI: Body mass index
FPG: Fasting plasma glucose
SBP: Systolic blood pressure
DBP: Diastolic blood pressure
TC: Total cholesterol
HDL-C: High density lipoprotein cholesterol
HOMA-IR: Homeostatic model assessment for insulin resistance
DII: Dietary inflammatory index
DM: Diabetes mellitus
IR: Insulin resistance
CVD: Cardiovascular diseases
Q1: First quartile
Q2: Second quartile
Q3: Third quartile
Q4: Fourth quartile
T1: First tertile
T2: Second tertile
T3: Third tertile
OR: Odds ratio
CI: Confidence interval
SD: Standard deviation
