# Supplementary material for: Associations between dietary inflammatory index and stroke risk: based on NHANES 2005–2018
Source: Sci Rep. 2024 Mar 20;14:6704. doi: 10.1038/s41598-024-57267-9 (PMC10954724; doi:10.1038/s41598-024-57267-9)
Supplement: Supplementary file 1 — Supplementary Tables. [file 41598_2024_57267_MOESM1_ESM.docx]

**Associations Between Dietary Inflammatory Index and the Prevalence of Stroke: based on NHANES 2005-2018**

Ruixian Huang ^a†^, Fengxia Lai ^a†^, Le Zhao ^a^, Jingjing Zhang ^a^, Hao Chen ^a^, Shuang Wang ^a^, Canjin Chen ^a^, Wenhao Wang ^a^, Zhenhua Mai ^a,b,*^, Yuanlin Ding ^a,*^, Danli Kong ^a,*^

^a^ Department of Epidemiology and Medical Statistics, School of Public Health, Guangdong Medical University, Dongguan, 523808, China.

^b^ Department of Critical Care Medicine, Affiliated Hospital of Guangdong Medical University

Zhanjiang, 524001, China.

**^*^ Correspondence:** Danli Kong: gdmckdl@163.com; Yuanlin Ding: gdmudyl@163.com; Zhenhua Mai: zhenhuamai@gdmu.edu.cn;

**^†^ Co-author:** The authors have the same contribution.

**Supplementary Table S1** Characteristics of the participants according to the tertiles of DII ^*^.

| Variable | Tertiles of DII | | | *χ*^2^/*t* | *P* value |
| --- | --- | --- | --- | --- | --- |
|  | T1 | T2 | T3 |  |  |
| Age | 47.71 ± 16.07 | 47.37 ± 16.96 | 47.09 ± 17.55 | -1.14 | 0.258 |
| Gender |  |  |  | 164.50 | <0.001 |
| Female | 1,699 (0.39) | 2,172 (0.51) | 2,637 (0.62) |  |  |
| Male | 2,656 (0.61) | 2,182 (0.49) | 1,717 (0.38) |  |  |
| Race |  |  |  | 12.41 | <0.001 |
| Non-Hispanic Black | 644 (0.07) | 850 (0.11) | 1,053 (0.14) |  |  |
| Non-Hispanic White | 2,021 (0.72) | 1,919 (0.68) | 1,890 (0.67) |  |  |
| Mexican American | 771 (0.09) | 684 (0.08) | 625 (0.08) |  |  |
| Other Hispanic | 396 (0.05) | 466 (0.05) | 452 (0.06) |  |  |
| Other race | 523 (0.07) | 435 (0.07) | 334 (0.06) |  |  |
| Education level |  |  |  | 49.84 | <0.001 |
| Less than high school | 818 (0.12) | 1,013 (0.15) | 1,260 (0.20) |  |  |
| Completed high school | 836 (0.18) | 998 (0.24) | 1,167 (0.29) |  |  |
| More than high school | 2,701 (0.70) | 2,343 (0.61) | 1,927 (0.51) |  |  |
| Married |  |  |  | 12.54 | <0.001 |
| Never married | 700 (0.16) | 772 (0.18) | 817 (0.19) |  |  |
| Partner or married | 2876 (0.70) | 2,645 (0.63) | 2,439 (0.60) |  |  |
| Separated or divorced | 532 (0.10) | 606 (0.13) | 698 (0.15) |  |  |
| Widowed | 247 (0.04) | 331 (0.06) | 400 (0.07) |  |  |
| Drinking status |  |  |  | 19.75 | <0.001 |
| Never | 488 (0.08) | 553 (0.11) | 716 (0.13) |  |  |
| Former drinker | 598 (0.11) | 709 (0.13) | 845 (0.16) |  |  |
| Current drinker | 3,269 (0.81) | 3,092 (0.76) | 2,793 (0.70) |  |  |
| BMI | 28.46 ± 6.59 | 29.04 ± 6.91 | 29.61 ± 7.16 | 5.38 | <0.001 |
| Energy intake | 2495.68 ± 788.98 | 2075.52 ± 668.32 | 1647.81 ± 595.35 | -30.03 | <0.001 |
| FPG | 5.87 ± 1.65 | 5.88 ± 1.62 | 5.91 ± 1.65 | 0.49 | 0.625 |
| Insulin | 11.90 ± 14.93 | 13.05 ± 16.14 | 12.85 ± 12.77 | 2.42 | 0.017 |
| SBP | 120.81 ± 16.13 | 121.83 ± 16.45 | 122.04 ± 18.02 | 2.90 | 0.004 |
| DBP | 70.30 ± 11.40 | 70.00 ± 12.08 | 68.59 ± 12.65 | -3.84 | <0.001 |
| TC | 4.95 ± 1.03 | 5.04 ± 1.08 | 4.99 ± 1.10 | 2.33 | 0.022 |
| HDL-C | 1.41 ± 0.42 | 1.41 ± 0.43 | 1.38 ± 0.41 | -1.31 | 0.194 |
| HOMA-IR | 3.37 ± 6.01 | 3.67 ± 5.89 | 3.61 ± 5.42 | 1.80 | 0.074 |
| Quartiles of HOMA-IR |  |  |  | 6.72 | <0.001 |
| Q1 | 1,210 (0.31) | 1,065 (0.27) | 992 (0.25) |  |  |
| Q2 | 1,115 (0.26) | 1,065 (0.25) | 1,085 (0.26) |  |  |
| Q3 | 1,053 (0.23) | 1,091 (0.25) | 1,121 (0.25) |  |  |
| Q4 | 977 (0.20) | 1,133 (0.23) | 1,156 (0.24) |  |  |
| Stroke |  |  |  | 8.63 | <0.001 |
| No | 4,239 (0.98) | 4,176 (0.97) | 4,140 (0.96) |  |  |
| Yes | 116 (0.02) | 178 (0.03) | 214 (0.04) |  |  |
| DM |  |  |  | 14.34 | <0.001 |
| No | 3,568 (0.87) | 3,408 (0.84) | 3,343 (0.82) |  |  |
| Yes | 787 (0.13) | 946 (0.16) | 1,011 (0.18) |  |  |
| Hypertension |  |  |  | 3.11 | 0.031 |
| No | 2,609 (0.64) | 2,506 (0.62) | 2,377 (0.60) |  |  |
| Yes | 1,746 (0.36) | 1,848 (0.38) | 1,977 (0.40) |  |  |
| Hyperlipidemia |  |  |  | 3.75 | 0.026 |
| No | 1,311 (0.31) | 1,162 (0.28) | 1,117 (0.27) |  |  |
| Yes | 3,044 (0.69) | 3,192 (0.72) | 3,237 (0.73) |  |  |
| Anti-diabetic Drugs |  |  |  | 5.12 | 0.007 |
| No | 3,905 (0.92) | 3,821 (0.91) | 3,799 (0.90) |  |  |
| Yes | 450 (0.08) | 533 (0.09) | 555 (0.10) |  |  |
| Anti-hypertensive Drugs |  |  |  | 3.61 | 0.031 |
| No | 3,052 (0.74) | 2,915 (0.72) | 2,853 (0.70) |  |  |
| Yes | 1,303 (0.26) | 1,439 (0.28) | 1,501 ((0.30) |  |  |
| Anti-hyperlipidemic Drugs |  |  |  | 0.27 | 0.747 |
| No | 3,468 (0.81) | 3,419 (0.81) | 3,441 (0.82) |  |  |
| Yes | 887 (0.19) | 935 (0.19) | 913 (0.18) |  |  |

^*^ ~~Rate~~ Percentage and mean ± standard deviation were weighted. The linear regression was used for continuous variable and *χ*^2^ test was used for categorical variables.

**Supplementary Table S2** Risk of stroke according to tertiles of DII.

|  | Model 1 ^a^ | | Model 2 ^b^ | | Model 3 ^c^ | |
| --- | --- | --- | --- | --- | --- | --- |
|  | *OR* (95% *CI*) | *P* value | *OR* (95% *CI*) | *P* value | *OR* (95% *CI*) | *P* value |
| Tertiles of DII |  |  |  |  |  |  |
| T1 | Ref. (1.00) |  | Ref. (1.00) |  | Ref. (1.00) |  |
| T2 | 1.54 (1.14-2.07) | 0.005 | 1.39 (0.98-1.97) | 0.064 | 1.39 (0.98-1.98) | 0.064 |
| T3 | 1.90 (1.44-2.50) | <0.001 | 1.46 (1.01-2.11) | 0.041 | 1.46 (1.01-2.11) | 0.040 |
| *P*-trend | <0.001 |  | 0.049 |  | 0.048 |  |
| For 1-SD increase | 1.42 (1.27-1.58) | <0.001 | 1.31 (1.13-1.51) | <0.001 | 1.31 (1.13-1.51) | <0.001 |

^a^ Model 1: Did not adjust any covariates;

^b^ Model 2: Adjusted for age, gender, race, education, marital status, BMI, energy intake, drinking status, FPG, SBP, DBP, TC, HDL-C, DM, hypertension, hyperlipidemia, antidiabetic drugs, antihypertensive drugs, and antihyperlipidemic drugs;

^c^ Model 3: Further adjusted for HOMA-IR.

**Supplementary Table S3** Risk of stroke by tertiles of dietary inflammatory index stratified according to HOMA-IR

|  | Participants / Event | Model 1 ^a^ | Model 2 ^b^ |
| --- | --- | --- | --- |
| Tertiles of DII |  |  |  |
| 0.03 ≤ HOMA-IR < 1.50 |  |  |  |
| T1 | 1,210/26 | Ref. (1.00) | Ref. (1.00) |
| T2 | 1,065/31 | 2.13 (1.07-4.22) | 1.94 (0.85-4.44) |
| T3 | 992/45 | 2.23 (1.23-4.05) | 2.04 (0.90-4.65) |
| *P*-trend |  | 0.004 | 0.074 |
| 1.50 ≤ HOMA-IR < 2.51 |  |  |  |
| T1 | 1,115/26 | Ref. (1.00) | Ref. (1.00) |
| T2 | 1,065/36 | 1.28 (0.68-2.42) | 1.19 (0.58-2.41) |
| T3 | 1,085/47 | 1.56 (0.84-2.89) | 1.20 (0.52-2.79) |
| *P*-trend |  | 0.155 | 0.679 |
| 2.51 ≤ HOMA-IR < 4.36 |  |  |  |
| T1 | 1,053/28 | Ref. (1.00) | Ref. (1.00) |
| T2 | 1,091/46 | 1.33 (0.75-2.34) | 1.17 (0.69-1.99) |
| T3 | 1,121/53 | 2.00 (1.20-3.31) | 1.61 (0.93-2.77) |
| *P*-trend |  | 0.008 | 0.087 |
| HOMA-IR ≥ 4.36 |  |  |  |
| T1 | 997/36 | Ref. (1.00) | Ref. (1.00) |
| T2 | 1,133/65 | 1.38 (0.80-2.38) | 1.34 (0.77-2.32) |
| T3 | 1,156/69 | 1.66 (1.00-2.77) | 1.41 (0.82-2.42) |
| *P*-trend |  | 0.048 | 0.235 |
| *P_interaction_* |  | 0.735 | 0.628 |

^a^ Model 1: Did not adjust any covariates;

^b^ Model 2: Adjusted for age, gender, race, education, marital status, BMI, energy intake, drinking status, FPG, SBP, DBP, TC, HDL-C, DM, hypertension, hyperlipidemia, antidiabetic drugs, antihypertensive drugs, and antihyperlipidemic drugs.

**Supplementary Table S4** The details about the quartiles of DII, HOMA-IR and the tertiles of DII between depressed group and non-depressed group.

|  | Stroke | |
| --- | --- | --- |
|  | No (*n* = 12547) | Yes (*n* = 508) |
| Quartiles of HOMA-IR |  |  |
| Q1 | [0.028-1.492] | [0.035-1.750] |
| Q2 | (1.492-2.490] | (1.750-2.989] |
| Q3 | (2.490-4.323] | (2.989-5.261] |
| Q4 | (4.323-269.41] | (5.261-86.743] |
| Quartiles of DII |  |  |
| Q1 | [-4.680-0.234] | [-3.246-0.909] |
| Q2 | (0.234-1.770] | (0.909-2.318] |
| Q3 | (1.770-3.003] | (2.318-3.489] |
| Q4 | (3.003-5.502] | (3.489-4.970] |
| Tertiles of DII |  |  |
| T1 | [-4.680-0.805] | [-3.246-1.444] |
| T2 | (0.805-2.597] | (1.444-2.995] |
| T3 | (2.597-5.502] | (2.995-4.970] |

Abbreviations: HOMA-IR, homeostatic model assessment for insulin resistance; DII, dietary inflammatory index; Q1, first quartile; Q2, second quartile; Q3, third quartile; Q4, fourth quartile; T1, first tertile; T2, second tertile; T3, third tertile.
